# Phenolic Profile, Antioxidant, and Antidiabetic Potential Exerted by Millet Grain Varieties

**DOI:** 10.3390/antiox9030254

**Published:** 2020-03-20

**Authors:** Fred Kwame Ofosu, Fazle Elahi, Eric Banan-Mwine Daliri, Ramachandran Chelliah, Hun Ju Ham, Joong-Hark Kim, Sang-Ik Han, Jang Hyun Hur, Deog-Hwan Oh

**Affiliations:** 1 Department of Food Science and Biotechnology, College of Agriculture and Life Sciences, Kangwon National University, Chuncheon, Gangwon-do 24341, Korea; ofosufk17@kangwon.ac.kr (F.K.O.); elahidr@gmail.com (F.E.); ericdaliri@yahoo.com (E.B.-M.D.); ramachandran865@gmail.com (R.C.); 2 Department of Biological Environment, College of Agriculture and Life Sciences, Kangwon National University, Chuncheon, Gangwon-do 24341, Korea; widehamboo@kangwon.ac.kr (H.J.H.); jhhur@kangwon.ac.kr (J.H.H.); 3 Erom, Co., Ltd., Chuncheon, Gangwon-do 24427, Korea; jhkim2@erom.co.kr; 4 Department of Medical Biotechnology, College of Biomedical Sciences, Kangwon National University, Chuncheon, Gangwon-do 24341, Korea; 5 Department of Southern Area Crop Science, NICS Upland Crop Breeding Res. Div., 181, Hyeoksin-ro, Iseo-myeon, Wanju-Gun, Jeollabuk-do 55365, Korea; han0si@korea.kr

**Keywords:** millet grains, antioxidant activities, phenolic compounds, flavonoids, digestive enzymes inhibitors, advanced glycation endproducts, functional food

## Abstract

This study evaluated the potential antioxidant and antidiabetic properties in vitro of four millet grain varieties cultivated in South Korea. The free fractions were tested for their total antioxidant capacity using 2,2′-azino-bis (3-ethylbenzothiazoline-6-sulfonic acid) diammonium salt (ABTS^+^) and 2,2′-diphenyl-1-picrylhydrazyl (DPPH) radical scavenging assays, followed by α-glucosidase, α-amylase, and advanced glycation endproducts (AGEs) formation inhibition assays. The total phenolics, flavonoids, and condensed tannins in the free fractions ranged from 107.8 to 136.4 mg ferulic acid equivalent (FAE)/100 g, 101.3 to 115.8 mg catechin equivalent (CE)/100 g, and 17.65 to 59.54 mg catechin equivalent (CE)/100 g, respectively. Finger Italian millet had the highest total phenolic content (136.4 mg FAE/100 g) and flavonoid content (115.8 mg CE/100 g). Barnyard and finger Italian millet showed the highest DPPH (IC_50_ = 359.6 µg/mL and 436.25 µg/mL, respectively) and ABTS radical scavenging activity (IC_50_ = 362.40 µg/mL and 381.65 µg/mL, respectively). Similarly, finger Italian millet also exhibited significantly lower IC_50_ values for the percentage inhibition of α-glucosidase (18.07 µg/mL) and α-amylase (10.56 µg/mL) as compared with acarbose (IC_50_ = 59.34 µg/mL and 27.73 µg/mL, respectively) and AGEs formation (33.68 µg/mL) as compared with aminoguanidine (AG) (52.30 µg/mL). All eight phenolic compounds identified in finger Italian millet were flavonoids, with flavanols being the predominant subclass. Taken together, millet flavonoids play important roles in the prevention and management of type 2 diabetes, and hence finger Italian millet has the potential to be developed as a functional food.

## 1. Introduction

Type 2 diabetes (T2D) is the most common type of diabetes, accounting for approximately 90% of all diabetes worldwide. T2D is characterized by hyperglycemia and abnormal carbohydrate metabolism, developed due to insulin resistance and pancreatic β-cell dysfunction [1,2]. Long-term exposure to high blood glucose levels has been implicated in the overproduction of reactive oxygen species (ROS). Excessive ROS-induced oxidative stress is one of the main mechanisms of the progression of diabetes causing cellular damage and resulting in various gene expression dysregulations which lead to impaired insulin secretion and insulin signaling [1,3,4]. The postprandial blood glucose levels have been found to play an important role in the onset and development of complications in T2D [5]. Hyperglycemia can also result in excessive non-enzymatic glycation of proteins and formation of advanced glycation endproducts (AGEs). The glycation modifications can further deteriorate the pathology of diabetes by contributing to nephropathy, cataracts, vasculopathy, and atherosclerosis [6]. Pancreatic α-amylase and intestinal α-glucosidase are key enzymes for dietary carbohydrate digestion and glucose release. One of the effective therapeutic strategies for controlling postprandial hyperglycemia is to retard the absorption of glucose through the inhibition of these enzymes. The prevalence of diabetes has necessitated an increased search for the development of natural remedies and dietary interventions, which are safer alternatives to synthetic drugs for the management of the disease. Growing evidence suggests that phytochemicals such as polyphenols from cereal grains and other medicinal plants offer potential therapeutic benefits such as alleviating diabetes and obesity complications, as well as inhibitory effects against α-amylase and α-glucosidase [7,8,9,10].

Millets are a collective term referring to several small-seeded edible grasses that belong to the family *Poaceae* (previously called *Gramineae*)*,* and are cultivated in arid and semiarid regions of the world [11]. Millets are naturally tolerant to most biotic and abiotic stresses and they are considered to be the sixth most important cereal in the world. They serve as a staple food in many African and Asian populations [12]. Millets are nutritious and provide considerable amounts of health benefits in multigrain and gluten-free cereal products [13]. In addition to nutritional benefits, millets contain numerous phytochemicals, mainly phenolic compounds, which can be useful in the management of metabolic disorders such as diabetes, cancer, and cardiovascular diseases [14]. These beneficial health outcomes could be due to the additive and synergistic effects of several compounds present in the grains, thus, their identification is of great importance. However, several factors such as genotype, soil, as well as environmental and climatic conditions affect the nutrient and phytochemical compositions of cereals [15,16,17]. It is worth noting that the inhibition of digestive enzymes depends on the phenolic content and also the individual phenolic type.

Despite increasing efforts to find natural potent inhibitors of advanced glycation formation to diminish their harmful consequences, investigations of millet phenolics are still limited. Therefore, it is necessary to evaluate the phenolic composition and bioactivities of millet varieties to explore their potential use as natural antioxidants and therapeutics for the development of functional foods. To the best of our knowledge, this study is the first to report potential antiglycation properties of millet grains, although several studies are available on medicinal plants, fruits, and vegetables. This study aimed to assess the antidiabetic activity in vitro of different millet cultivars grown in South Korea. The extracts prepared from millet (M), Italian millet (IM), barnyard millet (BM), and finger Italian millet (FIM) were used to compare their antioxidant and antidiabetic properties (α-amylase, α-glucosidase, and AGEs inhibition) in vitro. This study further investigated the phenolic composition using UHPLC-DAD-QTOF-MS^2^.

## 2. Materials and Methods

### 2.1. Chemicals and Reagents

The following liquid chromatography standards: gallic acid, caffeic acid, ferulic acid, p-coumaric acid, catechin, and quercetin were purchased from Sigma-Aldrich (Seoul, Korea). The enzymes α-amylase from *Bacillus licheniformis* (Chicago, IL, USA), α-glucosidase from *Saccharomyces cerevisiae*, acarbose, and aminoguanidine (AG) were all purchased from Sigma-Aldrich (Seoul, Korea). Sodium carbonate, potassium phosphate dibasic, potassium phosphate monobasic, sodium dihydrogen phosphate, sodium phosphate dibasic sodium chloride, soluble starch, 3,5-dinitrosalicyclic acid, 4-nitrophenyl α-D-glucopyranoside, Folin–Ciocalteu reagent, 2,2′-diphenyl-1-picrylhydrazyl (DPPH), 2,2′-azino-bis (3-ethylbenzothiazoline-6-sulfonic acid) diammonium salt (ABTS), 6-hydroxy-2,5,7,8-tetramethylchromane-2-carboxylic acid (Trolox), hexane, ethanol, methanol, sodium hydroxide, hydrochloric acid, sodium carbonate, and potassium persulfate were purchased. All other chemical reagents used were analytical grade.

### 2.2. Materials

**Figure 1:**
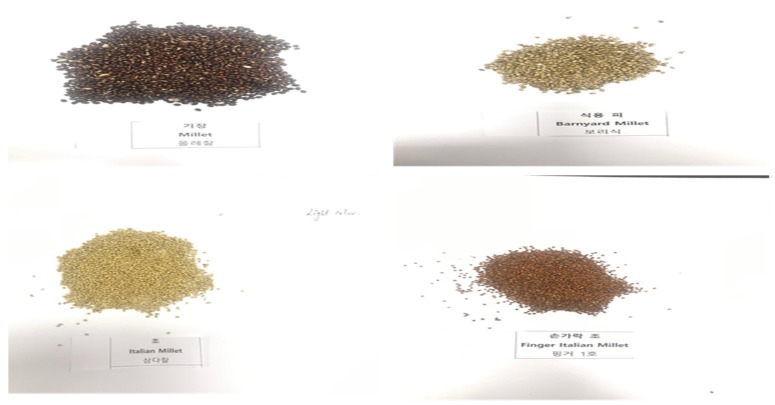
Pictures of millet grains used in the present study.

Whole millet grain varieties used in this study were provided by the Korean Rural Development Administration (KRDA) (Figure 1). Millet grains were cleaned and pulverized into fine powder using an electric mill and sieved through mesh 40. Samples were stored at −20 °C prior to extraction.

### 2.3. Preparation of Ethanolic Extracts

Soluble phenolic compounds were extracted as described by Pradeep and Sreerama with some modifications [7]. Samples were defatted with hexane using a soxhlet apparatus to remove lipids. Soluble phenolics from defatted samples (5 g) were extracted with 70% ethanol (1:20 w/v) in an orbital shaker for 1 h at 50 °C. After centrifugation at 4000× *g* for 10 min, the supernatant was collected, and the residue was re-extracted twice under the same conditions. The combined supernatants were concentrated under vacuum at 40 °C and freeze-dried. The lyophilized solids were stored at −20 °C and reconstituted in ethanol for further use.

### 2.4. Total Phenolic Content (TPC)

Total phenolic content (TPC) was measured as described by Ainsworth and Gillespie with slight modifications using a 24-well microplate with ferulic acid as the standard [18]. Briefly, 100 μL sample extracts, standard or 95% (v/v) methanol blank was added to 200 μL Folin–Ciocalteu reagent and vortexed thoroughly. The mixture was incubated at room temperature for 2 h after adding 800 μL of 700 mM sodium carbonate. The absorbance was read at 765 nm using a SpectraMax i3 plate reader (Molecular Devices Korea, LLC, Seoul, Korea). The total phenolic content was calculated from the ferulic acid standard curve and expressed as milligrams of ferulic acid equivalents per 100 g of sample (mg FAE/100 g).

### 2.5. Total Flavonoid Content (TFC)

Total flavonoid content (TFC) of ethanol extracts was determined using a 24-well microplate by the AlCl_3_ method described by Apea-Bah et al. [19] with some modifications. Briefly, 250 μL sample extracts, standard was added to 75 μL NaNO_2_ (50 g L^−1^) and 1 mL distilled water. After 5 min, 75 μL AlCl_3_ (100 g L^−1^) was added to the reaction mixture. After 6 min, 500 μL of 1 M NaOH and 600 μL distilled water was added. The absorbance at 510 nm was read after shaking for 30 s in a SpectraMax i3 plate reader (Molecular Devices Korea, LLC, Seoul, Korea). Catechin was used as a standard and results were expressed as milligram catechin equivalents per 100 g of sample (mg CE/100 g).

### 2.6. Total Condensed Tannin Content (CTC)

Total condensed tannin content (TCT) of the ethanol extract was measured by the vanillin assay with modification [20]. A sample solution (40 μL) was mixed with 4% vanillin reagent (200 μL) in a 96-well microplate. The absorbance of the mixture maintained at 30 °C was measured after 20 min at 500 nm against a blank solution, which was prepared according to the procedure above except that 4% vanillin reagent was substituted by 4% hydrochloric acid. Catechin was used as a standard and results were expressed as milligrams of catechin equivalents per gram of sample (mg CE/100 g) on dry weight basis.

### 2.7. Antioxidant Activities Assays

#### 2.7.1. DPPH Radical Scavenging Activity

DPPH assay was based on the method reported by Li et al. [21] with modification and using a 24-well microplate reader. Briefly, 200 μL sample extracts of various concentration was added to 2 mL freshly made 100 μM DPPH radical solution (4 mg DPPH in 100 mL 95% v/v methanol). Absorbance at 517 nm was read after 30 min of incubation at room temperature. The results were expressed in terms of IC_50_ representing the concentration of test extracts required to scavenge DPPH free radical by 50%.

#### 2.7.2. ABTS Radical Scavenging Activity

The method described by Xiang et al. [22] was used with slight modification. ABTS^+^ stock solution was prepared by reacting equal proportions of 7 mM ABTS solution with 2.45 mM potassium persulfate solution in the dark for 16 h at room temperature. The ABTS^+^ stock solution was diluted with ethanol to obtain an absorbance of approximately 0.70 at 734 nm. Appropriately, 80 μL sample extracts of various concentrations were added to 1 mL freshly prepared ABTS^+^ radical solution, and absorbance was read at 734 nm. The results were expressed in terms of IC_50_ representing the concentration of test extracts required to scavenge ABTS radical by 50%.

### 2.8. Carbohydrate Digestion Enzymes and Glycation Inhibitions

#### 2.8.1. α-Amylase Inhibitory Assay

The α-amylase inhibitory assay was adapted from Sekhon-Loodu and Rupasinghe, [9] with modification. Briefly, the test extracts, enzyme, and soluble starch were dissolved in 20 mM sodium phosphate buffer containing 6 mM NaCl (pH 6.9). The test extracts at different concentrations were dissolved in the buffer solution. To a test tube, 250 μL of pancreatic porcine α-amylase (1 U/mL, dissolved in the buffer (pH 6.9) and 100 μL of test extract at different concentrations were added. The mixture was pre-incubated at 37 °C for 15 min, before the addition of 250 μL of 0.5% starch. Then, the mixture was vortexed and incubated again at 37 °C for 20 min followed by the reaction termination using 1 mL of dinitrosalicylic acid color reagent. The tubes were placed in a heating block for 5 min, cooled to room temperature, and diluted with ultrapure water. Two hundred microliters of the reaction mixture were taken into a 96-well clear plate, and the absorbance was read at 540 nm using a SpectraMax i3 plate reader (Molecular Devices Korea, LLC, Seoul, Korea). The control was α-amylase without any inhibitor represented 100% enzyme activity. Appropriate test extract blanks containing the reaction mixture, except the enzyme, were used to correct for the color interference. A known α-amylase inhibitor, acarbose, was used for comparison studies. The percentage inhibition of the test sample on α-amylase was calculated as:

Inhibition (%) = 100 × (A_C_−A_S_)/A_C_

where A_C_ and A_S_ are the absorbance of control and test sample, respectively. The results were expressed in terms of IC_50_ representing the concentration of test extracts required to cause the enzyme inhibition by 50%.

#### 2.8.2. α-Glucosidase Inhibitory Assay

The α-glucosidase inhibitory assay was adapted from Sekhon-Loodu and Rupasinghe, [9] with modification. Briefly, various concentrations of freeze dried ethanolic extracts were prepared in 10 mM potassium phosphate buffer (pH 6.8). To a 24-well clear plate, a reaction mixture, containing 100 μL extract at different concentrations, 100 μL a-glucosidase (0.5 U/mL), and 300 μL of 10 mM potassium phosphate buffer (pH 6.8), were preincubated at 37 °C for 15 min before adding 100 μL of 5 mM p-nitrophenol- α-D-glucopyranoside substrate. Then, the mixture was incubated at 37 °C for the reaction to take place. After 15 min, 400 μL of stop solution containing 200 mM sodium carbonate was added. The absorbance at 405 nm was recorded using a SpectraMax i3 plate reader (Molecular Devices Korea, LLC, Seoul, Korea). Acarbose, a prescribed antidiabetic drug was used as a positive control. The control was the mixture of the enzyme and substrate without inhibitors, instead buffer was used. The sample blanks were the mixtures of test sample, substrate, and buffer except α-glucosidase. The inhibition (%) of the test sample on α-glucosidase was calculated similar to the α-amylase assay.

#### 2.8.3. Inhibition of AGEs Formation

The procedure used was adapted from Sekhon-Loodu and Rupasinghe, [9] with slight modifications. The final volume of the incubation mixtures was 1.0 mL, with equal volumes containing Bovine serum albumin (BSA, 5.0 mg/mL) and D-glucose (36 mg/mL), and negative control or the test samples or aminoguanidine at 0.01, 0.02, 0.04, 0.05, 0.1, 0.2, 0.5, and 1.0 mg/mL concentrations. Aminoguanidine, a known AGE formation inhibitor, was used as a positive control. All these solutions were dissolved in 0.2 M phosphate buffer saline (pH 7.4) containing sodium azide (0.02% w/v). The mixtures in Eppendorf tubes were incubated in triplicate at 37 °C for a week. Fluorescent AGEs were monitored on a microplate reader using 340 and 420 nm as the excitation and emission wavelengths. Experiments were conducted in triplicates. The percentage of the AGE inhibition was calculated as shown below:

$$\mathrm{Inhibition}\;\left(\%\right)=\left[1-\left(\frac{Fluorescent\;of\;the\;test}{Fluorescent\;of\;control}\right)\right]\times 100$$

The results were expressed in terms of IC_50_ representing the concentration of test extracts required to cause the inhibition of AGE formation by 50%.

### 2.9. Ultra-High PerformanceLliquid Chromatography Quadrupole Time-of-Flight Mass Spectrometry (UHPLC-Q-TOF-MS/MS) Phenolic Compounds Identification

An UHPLC (SCIEX ExionLC AD system, MA, USA) equipped with a controller, AD pump, degasser, AD autosampler, AD column oven, and photodiode array (PDA) detector (ExionLC) coupled to a quadrupole time-of-flight mass spectrometer (Q-TOF-MS) (X500_R_ QTOF) was used for UHPLC and mass spectrometric analyses (LC-MS^⁠2^). The protocol by Xiang et al. [22] was used with slight modification. The analytical column was a 100 × 3 mm, Accucore C⁠18 column (Thermo Fisher Scientific, Waltham, Massachusetts, USA). A sample (10 μL) was injected by an autosampler and eluted through the column with a binary mobile phase consisting of A (water containing 0.1% formic acid) and B (methanol), and the flow rate of 0.4 mL/min was used. A 25 min linear gradient was programmed as follows: 0–3.81 min, 9% to 14% B; 3.81–4.85 min, 14% to 15% B; 4.85–5.89 min, 15% B; 5.89–8.32 min, 15% to 17% B; 8.32–9.71 min, 17% to 19% B; 9.71–10.40 min, 19% B; 10.40–12.48 min, 19% to 26% B; 12.48–13.17 min, 26% to 28% B; 13.17–14.21 min, 28% to 35% B; 14.21–15.95 min, 35% to 40% B; 15.95–16.64 min, 40% to 48% B; 16.64–18.37 min, 48% to 53% B; 18.37–22.53 min, 53% to 70% B; 22.53–22.88 min, 70% to 9% B; 22.88–25.00 min, 9% B. Phenolic compounds were identified by comparing retention time (RT) and UV spectra information, and confirmed by ultra-high performance liquid chromatography quadrupole time-of-flight mass spectrometry (UHPLC-Q-TOF-MS^⁠2^, SCIEX, Framingham, MA, USA). The Q-TOF-MS was calibrated for the negative mode through the mass range of 100–1000 with a resolution of 5000. Full mass spectra were recorded using a capillary voltage of 1.45 kV and a cone voltage of 30 V. The flow rates of the cone gas (He) and desolvation gas (N_⁠2_) were 45 and 900 L/h, respectively. The desolvation gas temperature was set at 250 °C, the ion source temperature was 120 °C, and the collision energies of 15, 20, and 30 V were set to acquire the MS^⁠2^ spectra. Phenolic compounds were quantified based on the area of the peak at wavelengths of 280 nm for catechin, and quercetin, and 320 nm for *p*-coumaric acid, caffeic acid, gallic acid, and ferulic acid. The content of individual phenolics was expressed as µg/100g.

### 2.10. Statistical Analysis

The data were analyzed using Graphpad Prism 8.0. The differences of mean values among different millet phenolic fractions was determined using one-way analysis of variance (ANOVA) followed by Tukey’s test at *p* < 0.05 significance level. The results were presented as mean ± standard deviation (SD).

## 3. Results and Discussion

### 3.1. TPC, TFC, and CTC of Ethanol Extracts

Polyphenols are the main antioxidants that directly contribute to the antioxidant capacity of cereals [17]. Soluble TPCs of the four millet varieties, millet (M), Italian millet (IM), barnyard millet (BM), and finger Italian millet (FIM) are shown in Table 1. Soluble TPC ranged from 107.8 to 136.4 mg ferulic acid equivalent/100 g, DW. FIM soluble TPC was the highest, however, there was no significant difference between TPC of M, BM, and FIM. Italian millet contained significantly lower (*p* < 0.05) soluble TPC. Flavonoids are important antioxidants which contribute to the reduction of risk of chronic diseases [23]. From Table 2; Table 3, the free phenolic fraction was mainly composed of different flavonoids, with no phenolic acids identified. This is in accordance with literature, which reports that phenolic acids are mainly present in the bound fractions and flavonoids constitute the major class of free phenolic fractions [22,24]. TFC in free fractions of millet varieties ranged from 101.3 to 115.8 mg CE/100g. Finger Italian millet TFC was the highest, although there was no significant difference (*p* < 0.05) among these four varieties. The TPC and TFC of millets investigated in this study were higher than those previously reported by Pradeep and Sreerama [25]. Condensed tannins have demonstrated antioxidant, anti-inflammatory, antiviral, and antibacterial properties and are composed of flavan-3-ol units. In this study, BM (59.54 mg CE/100g, DW) and M (50.50 mg CE/100g, DW) varieties showed significantly higher (*p* < 0.05) CTC as compared with IM (36.37 mg CE/100g, DW) and FIM (17.65 mg CE/100g, DW). This result is inconsistent with that reported by Chandrasekara and Shahidi [11]. These authors found CTC in finger millet to be significantly higher than those in pearl, proso, foxtail, kodo, and little millets. This could be due to the genotype and cultivation environment and climatic conditions.

### 3.2. Antioxidant Capacity of Ethanol Extracts

**Figure 2:**
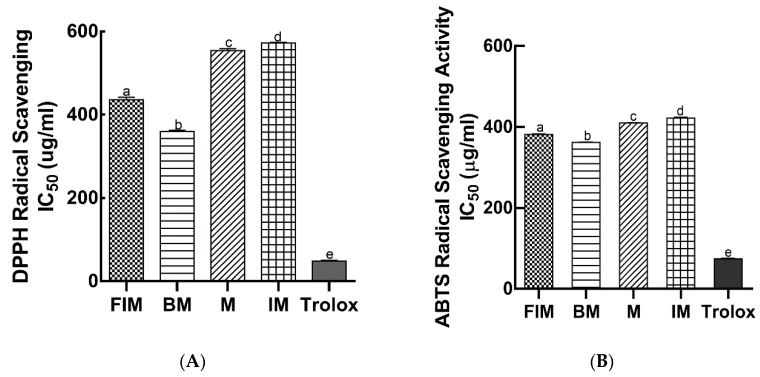
Antioxidant capacities of ethanol extracts of millet varieties. (**A**) 2,2′-Diphenyl-1-picrylhydrazyl (DPPH) radical scavenging activity IC_50_ values; **(B)** 2,2′-Azino-bis (3-ethylbenzothiazoline-6-sulfonic acid) diammonium salt (ABTS) radical scavenging activity IC_50_ values. FIM, finger Italian millet; BM, barnyard millet; M, millet; IM, Italian millet. Different lower case letters denote significant difference (*p* < 0.05).

Phenolic compounds in plants play key roles in protecting the body from oxidative stress, diabetes, cancer, and cardiovascular diseases. Phenolic compounds in various foods have been reported to significantly contribute to their antioxidant activities [24]. Therefore, plant-based therapeutics are potential alternatives to explore due to their safety and nutraceutical benefits [26]. To compare the antioxidant capacity of ethanol extracts of the four millet varieties, DPPH and ABTS radical scavenging assays were performed (Figure 2A,B). Among the millet grains evaluated, barnyard and finger Italian millet exhibited the highest DPPH radical scavenging activity with IC_50_ values of 359.6 and 436.25 µg/mL, respectively. Millet and Italian millet had a significantly lower DPPH radical scavenging activity with IC_50_ values of 554.3 and 572.9 µg/mL, respectively as compared to Trolox (48.7 µg/mL), a standard antioxidant compound. Similarly, barnyard millet (362.40 µg/mL) and finger Italian millet (381.65 µg/mL) showed the highest ABTS radical scavenging activity with lower IC_50_ values. Consistent with DPPH radical scavenging activity, millet and Italian millet had a significantly lower ABTS radical scavenging activity with IC_50_ values of 410.35 and 422.35 µg/mL, respectively as compared with Trolox (74.60 µg/mL). Our results are consistent with previous findings which demonstrated strong antioxidant activities from soluble phenolic fractions of millet varieties in vitro [22,24]. 

### 3.3. Antidiabetic Activity In Vitro

**Figure 3:**
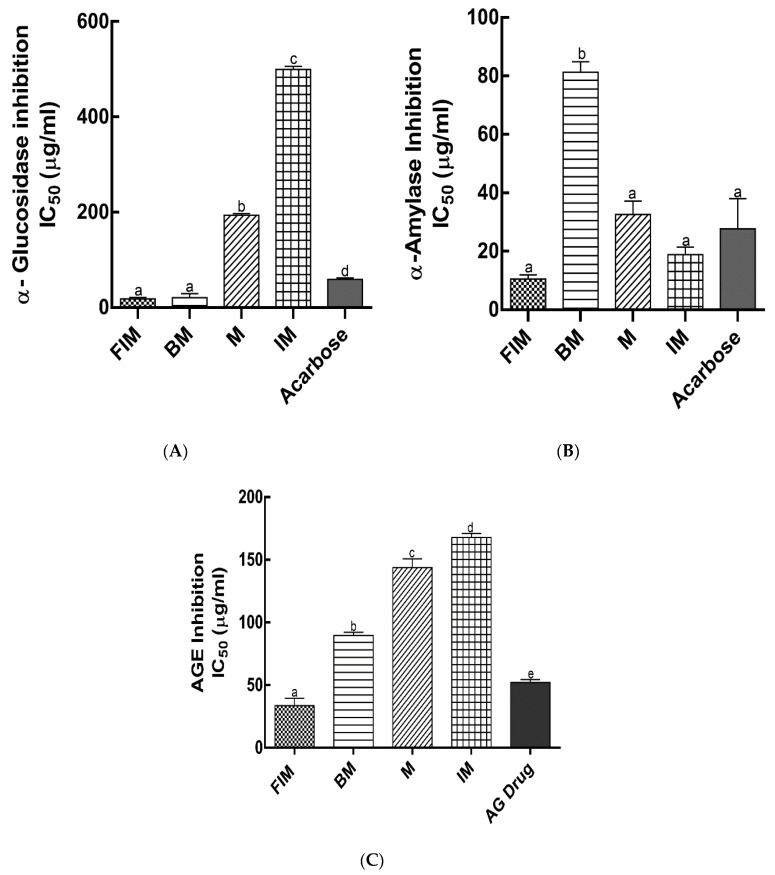
Digestive enzymes and advanced glycation endproducts inhibitory activities from ethanol extracts of millet varieties. (**A**) α-glucosidase inhibitory activity IC_50_ values; (**B**) α-amylase inhibitory activity IC_50_ values; (**C**) and advanced glycation endproducts (AGEs) inhibitory activity IC_50_ values. FIM, finger Italian millet; BM, barnyard millet; M, millet; IM, Italian millet. Different lower case letters denote significant difference (*p* < 0.05).

In the management of T2D, the primary factor to control is the postprandial blood glucose level. In addition to their antioxidant effects, dietary polyphenols have been shown to exert antihyperglycemic effects by competitive inhibition of digestive enzymes and binding to glucose transporters [27,28]. α-Amylase and α-glucosidase are carbohydrate hydrolyzing enzymes responsible for dietary starch digestion and oligosaccharides degradation to glucose, resulting in postprandial glucose increase. Therefore, of all the available antidiabetic therapeutic approaches, inhibition of α-amylase and α-glucosidase activities is considered to be one of the primary preventive strategies to manage T2D. The inhibition of α-amylase, α-glucosidase activity and AGEs formation (IC_50_ values) by ethanol extracts of millet varieties was investigated in this study (Figure 3A–C). Lower IC_50_ values indicated stronger inhibition activity of digestive enzymes and AGEs formation. Finger Italian millet and barnyard millet ethanol extracts exhibited the highest inhibition of α-glucosidase with IC_50_ values of 18.07 ± 3.27 µg/mL and 20.90 ± 7.46 µg/mL, respectively, which was significantly effective as compared with the commonly prescribed standard drug, acarbose IC_50_ value (59.34 ± 3.07 µg/mL). However, millet and Italian millet had a significantly lower α-glucosidase inhibition with IC_50_ values of 193.85 ± 3.63 µg/mL and 499.76 ± 5.46 µg/mL, respectively (Figure 3A). The enzyme inhibition activity of polyphenols has been reported to occur through nonspecific binding. Polyphenols show more effective α-glucosidase inhibition with an increase in molecular weight and degree of polymerization [29]. Millet extracts showed more potent inhibitory activity against α-glucosidase as compared with α-amylase [25]. However, our findings suggest the contrary which could be due to the environmental conditions, or the genotype which affects the composition and concentration of individual phenolic compounds. Finger Italian millet ethanol extracts exhibited the highest inhibition of α-amylase with IC_50_ values of 10.56 ± 1.43 µg/mL. However, this was not significantly different (*p* < 0.05) from Italian millet (18.89 ± 2.57 µg/mL) and millet (32.59 ± 4.61 µg/mL). Nonetheless, barnyard millet showed the lowest inhibition of α-amylase with IC_50_ value of 81.32 ± 3.54 µg/mL as compared with the drug, acarbose (27.73 ± 7.34 µg/mL) (Figure 3B). The soluble and bound phenolics (although soluble fractions showed higher inhibition) of foxtail and little millet cultivars were reported to have lower IC_50_ values as compared with the standard drug, acarbose [7,8]. This was similar to some of the millet varieties investigated in this study. Flavonoids are reported to show different inhibitory properties against α-glucosidase and α-amylase. Findings from Lim et al. [30] showed that the hydroxyl group at C3 of the C-ring and double bond between C2 and C3 on the C-ring of flavonoids play important roles in the inhibition of α-glucosidase and α-amylase, respectively. Recent studies have also reported that mechanisms of digestive enzymes inhibitory activities by phenolic compounds can involve their binding to amino acid residues at active sites of digestive enzymes via hydrogen bonding, thereby inhibiting the catalytic reaction of digestive enzymes on carbohydrates [31,32,33]. Our findings suggest that soluble millet phenolics, which are predominantly flavonoids, act as potent inhibitors of α-glucosidase and α-amylase, which could help reduce the release and absorption of glucose in the small intestine, and thus provide beneficial effects in diabetes by mitigating postprandial glycemic response.

The onset and progression of diabetes is linked to a key pathophysiological event, the formation of advanced glycation endproducts (AGEs). It is worth noting that increased efforts are being made to find natural compounds or products to diminish the deleterious effects of AGEs [34,35]. Recent studies have reported the potential antiglycation effects of polyphenols [36,37,38,39,40]. The ethanol extract of finger Italian millet exhibited the most significant inhibition of AGEs formation with IC_50_ value of 33.68 ± 5.98 µg/mL as compared with known antiglycation drug, aminoguanidine (IC_50_ = 52.30 ± 2.31 µg/mL). Barnyard millet, millet, and Italian millet extracts showed significantly lower inhibition of AGEs formation (IC_50_ = 89.76 ± 2.51, 143.85 ± 7.0, and 168.86 ± 3.19 µg/mL, respectively) as compared with the drug, aminoguanidine (IC_50_ = 52.30 ± 2.31 µg/mL) (Figure 3C). Growing evidence suggests that protein glycation inhibition by polyphenols strongly correlates to their antioxidant activity and phenolic content [41]. Phenolic fractions of medicinal plants showed stronger antiglycation properties than aminoguanidine [9]. The mechanisms by which polyphenols mitigate the deleterious consequences of advanced glycation involve the inhibition of (1) ROS formation, (2) Schiff base, (3) Amadori products and dicarbonyl formation, (4) the activation of detoxification enzyme system, and (5) the blocking interaction between AGEs and receptor [42]. 

### 3.4. Analyses of Phenolic Compounds by UHPLC-Q-TOF-MS^2^

Dietary phenolic compounds are considered to be the primary bioactive substances in various fruits, vegetables, beverages, and whole grains for the prevention of chronic diseases and improving health conditions. In the present work, the phenolic compositions of soluble ethanol extracts from finger Italian millet and barnyard millet were positively or tentatively identified by UHPLC-Q-TOF-MS^2^ in a negative mode. The mass and UV spectral data are summarized in Table 2 and Table 3. Phenolic identification and characterization were achieved by comparing the retention time (RT) with available authentic standards, UV spectra information, and confirmed by UHPLC-Q-TOF-MS^2^. Tentative compound identification was done with an online comprehensive dietary polyphenol database (Phenol-Explorer) [43]. A total of eight (8) and seven (7) phenolic compounds were tentatively identified from soluble extracts of finger Italian and barnyard millet, respectively, as shown in Table 2 and Table 3. All the phenolic compounds identified in finger Italian millet were flavonoids, with flavanols being the predominant subclass. Other phenolics belonging to different flavonoid subclasses included flavones, flavonols, isoflavonoids, dihydroflavonols, and their glycosides. Similarly, phenolics identified from barnyard millet soluble extracts were predominantly flavonoids with subclasses including flavanol, isoflavonoid, flavonols, and flavones. This finding is consistent with that reported by previous studies [22,24]. No phenolic acids were identified in the soluble ethanol extracts of both finger Italian and barnyard millet. Compound 2 ([M-H]^-^ *m/z* = 289.0721) was positively identified as catechin by comparing with a catechin standard, as can be seen in Table 2. Furthermore, Compounds 2 and 4 showed the same [M-H]^-^ at *m/z* 289.0721 suggesting the possibility of being an isomeric pair. Therefore, Compounds 3 and 4 were tentatively identified as catechin and epicatechin, respectively. Catechin and epicatechin have been previously reported in finger millet [22,24]. The remaining seven phenolics were all tentatively identified from Phenol-Explorer, an online polyphenol database (Table 2) [43].

Similarly, Compound 1 ([M-H]^−^ *m/z* = 289.0721) was positively identified as catechin by comparing with catechin standard, as can be seen in Table 3. Compounds 3 and 4 showed the same [M-H]^−^ at *m/z* 285.0408 indicating the possibility of being an isomeric pair. Therefore, Compounds 3 and 4 were tentatively identified as kaempferol and luteolin, respectively. The remaining six phenolics were all tentatively identified from the polyphenol database (Table 2). Two flavonoids, luteolin and tricin, were reported in Japanese barnyard millet [44]. To the best of our knowledge, we have reported for the first time the presence of formononetin, kaempferol, apigenin, isorhamnetin, and 3,7-dimethylquercetin in barnyard millet grown in South Korea. The quantitative results of the phenolic compounds in soluble fractions as compared with the calibration curve of available corresponding authentic standards are summarized in Table 4. The catechin content in finger Italian millet (577.49 µg/100g) was found to be four times higher as compared with their corresponding amount in barnyard millet (146.1 µg/100g). This could account for the higher inhibition activities of T2D related digestive enzymes, as catechins have been shown to be effective in controlling hyperglycemia and ameliorating diabetes [45,46]. However, this effect could also be synergistically enhanced by other flavonoids present. Catechin was reported as the predominant phenolic compound in finger millet in previous studies, however, catechin content in this study was lower as compared with earlier reports [22,24]. The remaining five standards were not present in the soluble fractions of finger Italian and barnyard millets. Phenolic acids of cereals are believed to be mostly present in bound form than in free fraction [11]. Phenolic acids have been reported as the predominant phenolics in bound fractions [22].

## 4. Conclusions

The present study provides new insights into the antioxidant and antidiabetic potential of selected Korean millet grains. Finger Italian millet and barnyard millet extracts exhibited high antioxidant capacity. The soluble phenolics of these millet varieties, predominantly flavonoids, demonstrated potent inhibition of α-glucosidase and α-amylase activities as compared with the commonly used drug, acarbose, indicating their potential to reduce postprandial hyperglycemia by retarding carbohydrate digestion. Furthermore, phenolic fractions, predominantly flavonoids, showed potent antiglycation properties, demonstrating their potential to diminish the deleterious consequences of AGEs. Therefore, further studies on animal models are needed to confirm the antidiabetic ability of these millet grains. Findings from this work are useful for the development of functional ingredients and foods for the prevention and management of diabetes and other chronic diseases.

## Figures and Tables

**Table 1:** Total phenolic content (TPC), total flavonoid content (TFC) and total condensed tannins content (CTC) of four millet grain varieties.

Millet Varieties	TPC (mg Ferulic Acid Equivalent/100 g, DW)	TFC (mg Catechin Equivalent/100 g, DW)	CTC (mg Catechin Equivalent/100 g, DW)
M	125.1 ± 6.36 ^a^	101.9 ± 9.1 ^a^	50.50 ± 3.83 ^a^
IM	107.8 ± 7.02 ^b^	105.4 ± 9.1 ^a^	36.37 ± 4.56 ^c^
BM	129.5 ± 4.95 ^a^	101.3 ± 10.4 ^a^	59.54 ± 4.63 ^a^
FIM	136.4 ± 7.07 ^a^	115.8 ± 9.1 ^a^	17.65 ± 3.95 ^b^

Results are expressed as mean ± SD. Different superscripts within each column denote significant difference (*p* < 0.05). M, millet; IM, Italian millet; BM, barnyard millet; FIM, finger Italian millet; DW, dry weight sample.

**Table 2:** Phenolic compounds identified in the ethanol extracts of finger Italian millet using ultra-high performance liquid chromatography quadrupole time-of-flight mass spectrometry (UHPLC-Q-TOF-MS^2^). RT, retention time.

Peak No.	RT (min)	Molecular Formula	Molecular Weight	[M-H]^-^ (m/z)	Compound Identified
1	8.73	C_21_H_24_O_11_	452.1321	451.1249	(+)-Catechin 3-O-glucose
2	9.54	C_15_H_14_O_6_	290.0797	289.0721	(+)-Catechin
3	9.67	C_21_H_22_O_12_	466.1113	465.1041	Dihydromyricetin 3-O-rhamnoside
4	11.06	C_15_H_14_O_6_	290.0797	289.0722	(−)-Epicatechin
5	12.42	C_15_H_12_O_7_	304.0585	303.0514	Dihydroquercetin
6	12.67	C_16_H_12_O_4_	268.0739	267.0664	Formononetin
7	15.63	C_15_H_10_O_6_	286.0482	285.0408	Kaempferol
8	16.42	C_15_H_10_O_5_	270.0530	269.0457	Apigenin

**Table 3:** Phenolic compounds identified in the ethanol extracts of barnyard millet by UHPLC-Q-TOF-MS^2^. RT, retention time.

Peak No.	RT (min)	Molecular Formula	Molecular Weight	[M-H]^−^ (m/z)	Compound Identified
1	9.54	C_15_H_14_O_6_	290.0797	289.0721	(+)-Catechin
2	12.68	C_16_H_12_O_4_	268.0739	267.0664	Formononetin
3	15.63	C_15_H_10_O_6_	286.0482	285.0408	Kaempferol
4	16.38	C_15_H_10_O_6_	286.0482	285.0408	Luteolin
5	16.43	C_15_H_10_O_5_	270.0530	269.0457	Apigenin
6	16.44	C_16_H_12_O_7_	316.0586	315.0513	Isorhamnetin
7	16.50	C_17_H_14_O_7_	330.0746	329.067	3,7-Dimethylquercetin

**Table 4:** Quantification of polyphenolic compounds in finger Italian and barnyard millet extracts by high performance liquid chromatography photodiode array (HPLC-PDA).

Compound	RT (min)	Finger Italian Millet (µg/100g)	Barnyard Millet (µg/100g)	Polyphenol Class
Gallic acid	2.18	ND	ND	Phenolic acid
Catechin	9.55	577.49	146.1	Flavonoid
Caffeic acid	10.15	ND	ND	Phenolic acid
p-Coumaric acid	11.95	ND	ND	Phenolic acid
Ferulic acid	12.58	ND	ND	Phenolic acid
Quercetin	15.3	ND	ND	Flavonoid

## Abbreviations

FIM	finger Italian millet
BM	barnyard millet
IM	Italian millet
M	millet
DPPH	2,2’-diphenyl-1-picrylhydrazyl
ABTS	2-azino-bis (3-ethylbenzothiazoline-6-sulfonic acid) diammonium salt
AGEs	advanced glycation endproducts
TPC	total phenolic content
TFC	flavonoid content
CTC	condensed tannin content
FAE	ferulic acid equivalent
CE	catechin equivalent
DW	dry weight
RT	retention time
UHPLC-Q-TOF-MS/MS	ultra-high performance liquid chromatography quadrupole time-of-flight mass spectrometry
